# The art of peer reviewing

**DOI:** 10.1007/s12024-024-00905-5

**Published:** 2024-10-29

**Authors:** Roger W. Byard

**Affiliations:** Adelaide School of Biomedicine, The University of Adelaide, Level 2, Room N237, Helen Mayo North, Frome Road, Adelaide, South Australia 5005 Australia

**Keywords:** Peer review, Publication, Writing, Assessment

## Abstract

Peer review of submitted manuscripts refers to the process of sending out papers for evaluation by suitably qualified academics/practitioners working in the same area. After their assessments and recommendations have been addressed by submitting authors editors will decide on whether publication is warranted or not. Unfortunately, ‘peer review’ has achieved a high status in courts without a real understanding of the way that the system works. Given that it has been deemed: ‘a flawed process, full of easily identified defects with little evidence that it works’, greater understanding of the nature of peer review is required. The following paper provides an overview of its strengths and weaknesses.

Do not discuss the manuscript with any of the co-authors - they may improve it. PD Home J R Coll Physicians Lond. 1988 [1].

One of the more enjoyable papers on how *not* to get your paper published was written by Home in 1988 [1]. The above quote is from that paper along with his conclusion that ‘it is the primary aim of many authors not to have their paper accepted for publication’. Many of his acerbic comments remain very true today. Certainly the process of scientific research and publishing has become more and more difficult in recent years with proliferation of both standard journals and so called predatory journals where anyone can publish a paper for a fee [2]. Artificial intelligence (AI) is increasingly being used to generate papers with assertions being made at international meetings that with AI papers can be finished in a matter of weeks and PhDs in less than a year. The concept of academic hypothesis testing and original writing appears to have been dealt a potentially lethal body blow. Peer review can now even be bypassed by posting research on online platforms, so called preprint servers.

Authorship has also become an issue of dubious validity with mega-authorship papers containing many hundreds of authors causing major elevations in the h-index of authors whose contributions are far from clear or substantive [3, 4]. The International Committee of Medical Journal Editors (ICMJE) clearly states that authorship should be based on: “1. Substantial contributions to the conception or design of the work; or the acquisition, analysis, or interpretation of data for the work; AND 2. Drafting the work or revising it critically for important intellectual content; AND 3. Final approval of the version to be published; AND 4. Agreement to be accountable for all aspects of the work in ensuring that questions related to the accuracy or integrity of any part of the work are appropriately investigated and resolved.” “All those designated as authors should meet all four criteria for authorship, and all who meet the four criteria should be identified as authors. Those who do not meet all four criteria should be acknowledged” [5]. As it is difficult to see how each of the 100 authors or more could fulfil these criteria, platforms such as ScholarGPS^®^ now do not include publications with more than 20 authors [6]. Perhaps authors who have knowingly participated in such potentially unethical activities should be penalised rather than rewarded?

One of the foundations of scientific publication over the years has been peer review; that is, the process of sending out papers that have been submitted to a journal for consideration for publication to suitably qualified academics/practitioners working in the same area. Following submission of a paper the usual process is for evaluation by the Editors-in-Chief to make sure that the paper is suited to the journal, is appropriately constructed with reasonable content, grammar and language, and meets ethical guidelines. If a paper passes this hurdle it will then be sent out to reviewers for comments and/or recommendations. From then on the situation becomes less clear as there are no standard guidelines for who exactly should conduct a review [7]. Most reviewers would have had no training in the review process and are unfortunately sometimes operating well outside their area of expertise. The irony is that the most expert individuals in a particular field are often the busiest and so the burden of review may fall to more junior, less-experienced colleagues. Increasingly experienced reviewers seem to be declining invitations from journals. Essentially the choice of reviewer falls to the editor who may also be dealing with a paper on a topic that is unfamiliar. Thus, there is no quality control for the review process although publishers do provide guidelines for authors on how to improve their submissions which may also be useful for reviewers [8]. Peer review has therefore been called ‘subjective’ and ‘inconsistent‘, a process that is based on ‘faith’ rather than ‘facts’ [7, 9].

Editors see many reviews of variable quality. The author has noted that some of the better writers are not very good at reviewing, while some of the best reviewers are not good at putting their own papers together – it is a very unpredictable process. Reviewers also vary greatly in their styles with some merely stating ‘accept’ or ‘reject’ without providing reasons for their decision, which is distinctly unhelpful to an editor. Other reviewers will obsessively concentrate on minor typographical or syntax corrections, something that is best left to the copyeditors if the paper is accepted. In the past good reviews used to provide a precis of a paper’s hypotheses, results and conclusions, to demonstrate that the reviewer had indeed read the work and had an understanding of what the authors were asserting. Such reviews are sadly becoming increasingly rare. One irritating habit that has developed is for reviewers to recommend publication or not directly to the author. This is not the reviewer’s role, but is the responsibility of the editors, based on an evaluation of sometimes multiple reviews. This is the reason for the separate box for reviewers to communicate their final recommendations privately to the editor and not to the author.

There are certain strategies that can assist a reviewer – such as checking the references first. Incorrectly cited references in a variety of styles not used by the journal signify to most experienced reviewers that the authors lack attention to detail and usually result in any areas in the text that are unclear being also assumed to be incorrect. Plagiarism is rife in certain disciplines and so a variety of different software is available to check for this.

Further evidence of problems with the review system occur when two often experienced reviewers assessing the same manuscript come up with diametrically opposed recommendations. As an editor it is often difficult to understand how this could happen. However, the process is to then to send the paper out to at least one other reviewer for a majority decision. It is sometimes unfortunate when an editor knows that a paper is worthy of publication but is over-ruled by external reviewers – but that is how the system works. Stories, some of which may be apocryphal, circulate in editorial circles of reviewers deliberately rejecting or delaying a paper’s publication as it represented rival research, or of taking the idea or method behind a paper and independently publishing it [7]. Some journals have a policy of identifying their reviewers to avoid this, however this can have the unfortunate result of having authors communicating their discontent directly to a reviewer. For this reason anonymity has an advantage. Other journals allow contributors to nominate reviewers who they consider would not provide an unbiased assessment of their work. Blinding reviewers to the identity of authors has not been found to improve the review process [7].

In conclusion, there is no doubt that reviewing is an art and not a science. To quote a disturbing assertion by Richard Smith, a former editor of the British Medical Journal: ‘peer review is a flawed process, full of easily identified defects with little evidence that it works’ [7]. Thus the next time that a lawyer in court obsesses about the importance of peer review feel free to quote this Commentary and perhaps ask for concrete (? peer reviewed) evidence that the peer review process has ever been shown to be valid. Could it be that we are dealing with yet another case of the Emperor’s new clothing?

## Key points

1. Peer review has been one of the foundations of scientific publishing.
2. This refers to the process of sending out papers for evaluation by suitably qualified academics/practitioners.
3. Although ‘peer review’ has achieved high status in courts there is a lack of understanding of the way in which the system works.
4. Peer review has been referred to as ‘a flawed process, full of easily identified defects with little evidence that it works’.
5. The process of peer review requires greater understanding of its strengths and weaknesses.

## Data Availability

None.
